# Insulin resistance in obese children and adolescents: from mechanisms to screening and exercise intervention

**DOI:** 10.3389/fped.2026.1679527

**Published:** 2026-02-02

**Authors:** Youxiang Cao, Rui Xu, Jie Zhang

**Affiliations:** 1 School of Physical Education, Nanjing Xiaozhuang University, Nanjing, China; 2 Nanjing Sport Institute, School of Sports and Health, Nanjing, China; 3 Laboratory of Kinesiology, Nanjing Sport Institute, Nanjing, China

**Keywords:** exercise, insulin resistance, mechanisms, obese children, prediction model

## Abstract

The early onset of obesity has become a serious public health problem and is accompanied by the low age range of various metabolic diseases. Obesity is strongly associated with the development of Insulin resistance (IR). IR is considered the pathological basis of several metabolic diseases and is associated with a high likelihood of future chronic disease in adulthood. But the onset of IR is highly insidious, and the early development of a predictive model of morbidity is essential to the prevention. Exercise can effectively prevent IR, but the optimal intensity and dose of exercise are controversial. Thus, this narrative review shows the mechanisms, screening, and exercise interventions of IR in obese children and adolescents.

## 1 Background

The increasing prevalence of obesity among children and adolescents has become a global public health problem. According to a study including 195 countries worldwide, 107 million children and adolescents were obese in 2015, and the prevalence of obesity among children and adolescents in over 70 countries has doubled since 1970 (1). Obesity can cause the body to produce a variety of metabolic diseases (2–4) and is a major cause of insulin resistance (IR) and prediabetes (5). The incidence of IR is 8.9% in children and adolescents with normal weights, 28.6% in overweight children and adolescents, and 44.3% in obese children and adolescents in China (6).

**Table 1:** The mechanism of obesity-induced insulin resistance.

Related mechanism	Roles in insulin resistance	References
Defects in insulin receptor substrate	Reverses insulin-induced phosphorylation in tyrosine residues of IRS-1 and so impairs insulin signal transduction	(32, 33, 147)
Disorders of glucose and lipid metabolism	Decrease in insulin receptor phosphorylation	(41–43)
Adipokines	Reduces the phosphorylation of the ISR1/2 tyrosine or inhibits islet β-cell function	(58, 60, 64)
Metabolic inflammation	Activation of IKKβ/NF-κB and JNK pathways, serine phosphorylation of IRS-1 in the site of 307	(69, 70, 72, 73)
Oxidative stress	Induces oxidative stress, impairs insulin signaling	(75–79)
Intestinal flora	Induces chronic inflammation and impairs insulin signaling	(82, 87)

**Table 2:** Studies that evaluated the performance of indicators in indentifying IR.

No.	Author	Study design	Sample	Predictors	Statistical analysis	Main results
1	Moreira et al.(148)	Transversal	*N* = 109, 7–11 years old, 31 normal, 23 overweight, and 55 obese children	BMI; WC; C index	ROC curve	AUC 0.90, 95%CI (0.83–0.97); 0.88, 95%CI (0.79–0.96); 0.78, 95%CI (0.66–0.90); 0.69, 95%CI (0.50–0.87)
2	Carneiro et al. (149)	Transversal	*N* = 148, 14.1 ± 2.1 years old, BMI: 23.7 ± 4.7 kg/m^2^	C index; WC; WHtR	ROC curve and logistic regression	High predictive accuracy for IR: (AUC 0.696, *p* = 0.008; 0.705, *p* = 0.004; 0.702, *p* < 0.001)
3	Manios et al. (150)	Transversal	*N* = 248 (109 boys), 10.46 ± 0.36 years old, BMI: 19.95 ± 3.89 kg/m^2^	BMI; WC; WHtR	Correlation and multiple linear regression	All high correlation with HOMA-IR (*p* < 0.001)
4	Hirschler et al. (151)	Transversal	*N* = 625 (31 boys), 9.6 ± 2.0 years old	WC; BMI	ROC curve and multiple logistic regression	AUC 0.728, 95%CI (0.74, 0.82); 0.77, 95%CI (0.73, 0.82)
5	Locateli et al. (152)	Transversal	*N* = 345 (120 boys), 15.4 ± 1.8 years old, overweight or obese	TyG index; TG/HDL-c; TyG index + TG/HDL-c	Spearman's correlation and ROC curve	TyG index: higher predictive accuracy for IR [AUC 0.743, 95%CI (0.690, 0.796)]
6	Kang et al. (153)	Transversal	*N* = 221 (168 boys), 11.1 ± 1.5 years old, overweight, and obese children	TyG index	ROC curve and Pearson's correlation	TyG index was well correlated with HOMA-IR (r = 0.41; *p* < 0.001)
7	Singhal et al. (154)	Transversal	*N* = 294 (134 boys), 14.9 ± 0.9 years old, BMI: 21.3 ± 3.8 kg/m^2^	Adiponectin	Multiple linear regression	High correlation with HOMA-IR [r = −1.4, 95%CI (−2.7, −0.1), *p* = 0.03]
8	Dikaiakou et al. (155)	Transversal	*N* = 367 (175 boys), 9.9 ± 2.3 years old, BMI: 27.4 ± 4.1 kg/m^2^	TyG index	ROC curve and multiple logistic regression and Pearson's correlation	AUC 0.65 which significantly differs from 0.5 (*p* < 0.001)
9	Rodríguez-Gutiérrez et al. (156)	Transversal	*N* = 1,006 (109 boys), 10.46 ± 0.36 years old, BMI: 19.95 ± 3.89 kg/m^2^	WC + acanthosis nigricans + pubertal status	ROC curve and multiple logistic regression	AUC 0.849, *p* < 0.05
10	Barchetta et al. (157)	Transversal	*N* = 909 (433boys), 10.3 ± 3.2 years old; BMI: 27.4 ± 4.4 kg/m^2^;	SPISE index	ROC curve and Pearson's correlation	Lower basal SPISE was an independent predictor of IGR development [OR = 3.89 (1.65–9.13), *p* = 0.002; ROC: 0.82 (0.72–0.92), *p* < 0.001].
11	Tantari et al. (158)	Transversal	*N* = 232 (105 boys), 13.2 (10.8–15.4) years olds, overweight and obesity	SPISE index	ROC curve	A SPISE cut-off ≤ 6.92 or ≤ 6.13 (based on the method used for insulin resistance detection), showed good sensitivity and specificity

AUC, area under curve; BMI, body mass index; CI, confidence interval; C index, conicity index; HDL-c, high-density lipoproteins cholesterol; HOMA-IR, homeostasis model assessment index of insulin resistance; IGR, impaired glucose regulation; ROC, receiver operating characteristic curve; SPISE index, single point insulin sensitivity estimator; TyG, triglyceride-glucose index; TC, total cholesterol; TG, Triglyceride; WC, waist circumference; WHtR, waist-to-height ratio.

IR is an abnormal state of decreased insulin sensitivity in body tissues, such as liver and muscle tissues, and is often considered the pathophysiological basis of metabolic diseases (7, 8). IR is not only extremely harmful to adults (9–12) but also to children and is usually accompanied by islet beta-cell dysfunction. The rate of pancreatic beta-cell dysfunction is faster in children and leads to related complications (13). In addition, IR during childhood and adolescence is closely associated with the development of cardiovascular diseases in adulthood (14). Therefore, diagnosing and preventing IR in obese children as early as possible is important. Risk prediction model and exercise intervention are effective means of preventing and treating insulin resistance in obese children.

This narrative review addresses the hazards, causes and mechanisms of insulin resistance in obese children, further summarizes the screening and the effects of exercise interventions on insulin resistance. The research presents a promising picture for the prevention and treatment insulin resistance in obese children.

## 2 Insulin resistance and health for children and adolescents

The adverse effects of IR are primarily due to the hyperinsulinemia (15), further lead to reduced insulin sensitivity, which represents an important risk factor for the development of type 2 diabetes (T2D) (16) and *β*-cell dysfunction (17). Besides hyperinsulinaemia, IR often associated with hyperlipidaemia, lipid metabolism disorders, oxidative stress and inflammation (18). In fact, IR has been demonstrated to be a reliable marker in the prediction of cardiovascular risk (19). According to the National Health and Nutrition Examination Survey (NHANES), IR is the most important risk factor for coronary heart disease in young people (20). When IR accured, it increases the risk of cardiovascular disease in the body through various mechanisms (21, 22).

IR not only seriously affects the health status of adults, but also has important harmful effects on children and adolescents, even to a more serious extent than adults. Unlike adults, when insulin sensitivity decreases in children and adolescents, pancreatic *β*-cells will disproportionately over-secrete insulin to maintain metabolic stability of the body (23), which puts the body in a long-term state of hyperinsulinemia (24); and hyperinsulinemia will be accompanied by an increase in appetite, which will further exacerbate obesity (25), forming a vicious circle. Compensated insulin overproduction also exacerbates oxidative stress, leading to rapid decline in *β*-cell function and ultimately irreversible damage to *β*-cell function (26, 27). Yaujnik et al. (14) also found that having IR in childhood leads to a significantly higher risk of developing cardiovascular disease in adulthood.

In addition, studies found that IR may be involved in the pathogenesis of atherosclerosis, based on evidence that the more pronounced the IR in adolescents, the higher the circulating biomarkers of endothelial dysfunction, and the lower the reduction in lipocalins, which act as anti-atherosclerotic agents (28). IR plays a key role in the pathogenesis of non-alcoholic fatty liver disease (NAFLD) by promoting lipogenesis in hepatocytes, impairing the inhibition of lipolysis and stimulating the secretion of adipokines and other cytokines (10). Clamping studies in adolescents and young adults suggest that NAFLD is associated with IR, possibly because of increased abdominal visceral obesity (16, 29). In conclusion, IR can be harmful to children and adolescents not only in the present, but also increase the risk of disease in adulthood (14).

## 3 The mechanisms of obesity-induced insulin resistance

### 3.1 Insulin receptor defects

**Figure 1:**
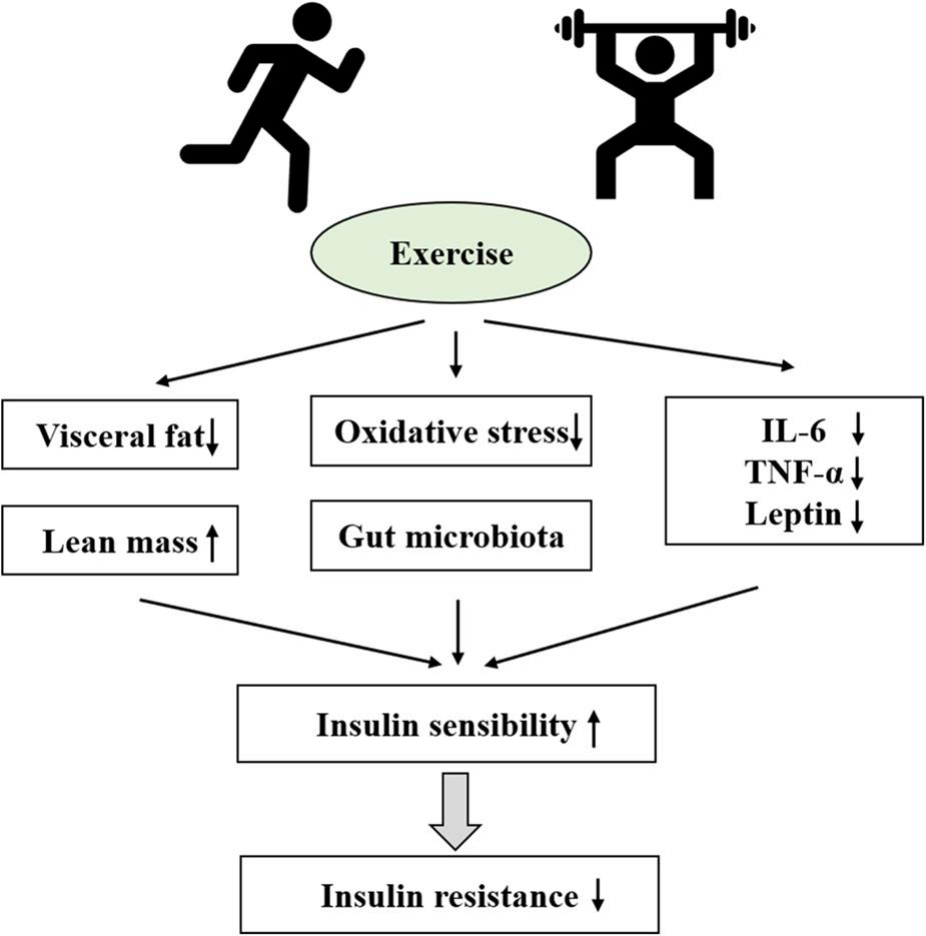
Schematic representation Nof obesity-induced insulin resistance. Unhealthy lifestyles (high fat diet or sedentary) can lead to obesity in normal weight children and adolescents. And obesity develops in children and adolescents can causes free fatty acids, IL-6, TNF-α, Leptin or adiponectin raised or oxidative stress. Eventually, this leads to insulin resistance. In addition, Obesity can also lead to the development of hyperlipidemia, hyperinsulinemia or meta-inflammation which promoted IR; And IR in turn promotes hyperlipidemia, hyperinsulinemia or meta-inflammation.

Insulin is an important hormone involved in the metabolism of sugars, lipids, and proteins in the human body. Its main physiological functions include promoting the absorption of blood glucose by body tissues, promoting the synthesis of glycogen, and inhibiting the breakdown of glycogen and gluconeogenesis (30). Insulin exerts its biological effects mainly through the phosphatidyl alcohol 3 kinase (PI3K) signaling pathway or the mitogen-activated protein kinase/Ras (MAPK/Ras) pathway (31). The PI3K molecule is composed of a 110 kD catalytic subunit and an 85 kD regulatory subunit, which also contains Src homology-2 (SH2) and Src homology-1 (SH1) structural domain. In this signaling pathway, insulin activates insulin receptor substrate (IRS) by binding to insulin receptor proteins on the cell surface, IRS binds to PI3K and activates it through a cascade effect, and activated PI3K binds to downstream protein kinase B (Akt), which mediates the transport of glucose out of the circulatory system through a series of reactions with glucose transporter protein 4 (GLUT4) (32). When IR occurs in the body, the PI3K signaling pathway of insulin is impaired, and the IRS cascade effect is blocked, preventing glucose from being absorbed and utilized by target tissues in a timely manner (33) (Figure 1).

### 3.2 Obesity

Miao et al. (5) found through a Mendelian randomization study that obesity is a strong causative factor for IR and prediabetes development in the body. Obesity leads to the enlargement of adipocytes and expansion of adipose tissues (34), which secrete a variety of adipokines involved in the body's metabolic and inflammatory processes. The endocrine dysregulation of adipose tissues may be an important factor for metabolic disorders in the body. In addition, obesity increases free fatty acid (FFA) content, facilitates mitochondrial fatty acid transport, and decreases *β*-oxidation capacity, thereby inhibiting fat oxidation (35), impairing glucose tolerance, and promoting the development of IR (36). Obesity can lead to the disorder of glucolipid metabolism, disorders of adiposity, oxygen stress, and inflammation response in the body, leading to the occurrence and development of IR under one or more factors (Table 1).

#### 3.2.1 Disorders of glucose and lipid metabolism

Obesity is usually closely related to glucose, lipid, and protein metabolism disorders in the body, such as elevated FFA (37) and branched chain amino acid (BCAA) levels (38, 39). Elevated FFA concentrations in obese individuals cause the enrichment of triglycerides, long-chain acylcarnitines, and diglycerides (DAGs) in cells or liver (40). In human skeletal muscles, elevated FFA levels lead to DAG enrichment and subsequent PKC activation (41), which in turn inhibits the phosphorylation of tyrosine sites on IRS-1/2 and subsequently leads to IR (42).

In addition, elevated FFA concentrations may increase the secretion of cytokines, such as tumour necrosis factor-alpha (TNF-α), interleukin-1 beta (IL-1β), and interleukin-6 (IL-6), leads to IR, through the activation of the nuclear factor kappa-B (NF-kB) pathway (43). The body becomes insulin resistant after 6 h of acute elevation of plasma FFA (44), and a decrease in FFA level significantly alleviates IR in the body (45). Elevated FFA level may be an important trigger for the transition from IR to T2DM (46–48). In addition, obesity-induced impaired intracellular lipid oxidation in skeletal muscles may be an important trigger for IR in the body (36).

#### 3.2.2 Adipokines

In recent years, a series of studies has confirmed that adipose tissue is not only an energy storage organ but also an endocrine organ and can secrete various bioactive factors (49). Numerous adipokines have been identified, including acute-phase cytokines such as C-reactive protein, plasminogen activator inhibitor 1, haptoglobin, TNF-α, IL-6, IL-8, IL-10, CC-motif chemokine ligand 2 (CCL2), CCL5, and C-X-C motif ligand 8 (CXCL8). Among these, leptin, adiponectin are closely associated with the occurrence and development of insulin resistance (50, 51). Lipocalin has a good insulin-sensitizing effect and has thus become a major subject of interest in studies about IR or T2DM due to obesity (52). Lipocalin and its receptor levels are significantly lower than normal levels in patients with IR due to obesity (53–55). In obese IR mice with reduced lipocalin levels due to diet or genetic defects, the exogenous injection of lipocalin improved insulin sensitivity (56), mainly because lipocalin acted through the activation of adenosine monophosphate-activated protein kinase (AMPK) phosphorylation (57). In conclusion, lipocalin can enhance insulin sensitivity and insulin function (58).

In addition to lipocalin, leptin is a protein molecule secreted mainly by adipocytes and plays a role in regulating body weight and body metabolism (59). It can regulate islet *β*-cell function directly (60) and indirectly through neuronal function (61). The leptin levels of children with IR caused by obesity are significantly higher than those in children with normal weights (62). However, in obese IR children, increased levels of endogenous or exogenous leptin no longer act to reduce body weight through inhibition possibly because of the reduced sensitivity of leptin-related action pathways in the hypothalamus or other central neurons, producing leptin resistance (63). Leptin resistance in the brain leads to the accumulation of triglycerides in the liver, blood, and pancreas, ultimately reducing insulin sensitivity and resulting in IR (64).

#### 3.2.3 Metabolic inflammation

Normal body cells or systems show inflammatory response to changes in homeostasis, including external bacterial stimuli and cell apoptosis, and physiological changes, such as excessive lipid accumulation (65). Inflammatory response is the basis of several pathophysiological responses (66). Low-intensity chronic inflammation caused by obesity is often called metabolic inflammation or meta-inflammation (67). The mechanisms of inflammation leading to IR mainly include inflammatory factors acting on the insulin signaling system to interfere with insulin receptors signal transduction. In addition, research also found that there is an inverse correlation between insulin sensitivity and plasma fibrinogen levels (68). Fibrinogen is frequently elevated in obesity, and reflecting the IL 6–mediated acute phase response, which associated with insulin resistance, endothelial dysfunction, and a prothrombotic profile, contributing to cardiometabolic risk.

TNF-α, secreted by adipocytes, is the most important one. It can contribute to IR by inhibiting glucose transporter type-4 (GLUT-4) expression in skeletal myocytes, cardiomyocytes, and adipocytes (69, 70), and its pathway activity leads to IR (71). In addition, elevated TNF-α levels activate stress-activated protein kinase (cJun-N-terminal-kinase, JNK), which inhibits the phosphorylation of IRS-1/2 leading to IR (72). C-reactive protein is another marker of inflammation associated with IR and metabolic diseases and is a widely used clinical biomarker. C-reactive protein could effect insulin sensitivity and glucose homeostasis through binds to leptin and block leptin signaling (73).

#### 3.2.4 Oxidative stress

Oxidative stress refers to the imbalance between the production and scavenging of oxygen free radicals in the body and leads to the excessive accumulation of reactive oxygen species (ROS) and resulting in damage to body tissues (74). Oxidative stress is closely associated with IR and can lead to IR by inhibiting insulin signaling pathway expression (75, 76), and obesity leads to oxidative stress followed by the stimulation of inflammatory factors through the IKKβ/NF-kB and JNK signaling pathways, thereby inducing IR (77, 78). In addition, oxidative stress inhibits PKB phosphorylation and GLUT-4 expression, ultimately leading to IR (79). In conclusion, the relationship between oxidative stress and IR is usually linked to inflammatory response.

#### 3.2.5 Intestinal flora

More than one trillion microorganisms exist in the human body and are mostly found in the gastrointestinal tract; these microorganisms are influenced by pH, oxygen, and nutritional status (80). Obesity decreases the proportion of *Akkermancia muciniphila* strains, which have a positive effect on insulin sensitivity (81). The intestinal flora regulates energy uptake and blood lipopolysaccharide levels and induces chronic inflammation leading to IR (82). In addition, microorganisms *in vivo* can cause IR by inducing increase in lipid level (82). In conclusion, the effect of the intestinal flora on T2DM and IR is exerted mainly through the induction of increases in inflammatory factor levels.

## 4 Screening and prevention

Currently, diagnostic methods for IR in children and adolescents have two main categories: precise measurement and indirect estimation methods. Precise measurement methods, such as hyperinsulinemic–euglycemic clamp technique (83) and intravenous glucose tolerance test (frequently sampled intravenous glucose tolerance test, FSIVGTT) combined with the micro-model mathematical analysis method (84). The hyperinsulinemic–euglycemic clamp technique is the gold standard for IR diagnosis (83) *in vivo*, and which involves infusion of insulin at a constant rate (determined by body size) while maintaining the glucose at a fixed concentration. But due to its test time and cost, this method is not widely used in clinical practice. The other type is the indirect estimation method, which is mainly related to fasting insulin, fasting glucose, and indices related to oral glucose tolerance test (OGTT). The commonly used diagnostic methods are homeostasis model assessment index of IR (HOMA-IR) (85), whole body insulin sensitivity index (WBISI) (86), and insulin sensitivity index (ISI) (87). One study on adolescents found that WBISI responds to insulin sensitivity changes earlier and better to the degree of IR than HOMA-IR (88).

However, direct and indirect measurement methods are *post hoc* diagnosis and cannot effectively provide early warning of IR in obese children and adolescents. In addition, IR is untimely diagnosed because the fasting blood sugar of the body remains in the normal range. Therefore, it is necessary to assess the risk of IR before it occurs through prediction model (89).

The current prediction of IR is mainly based on single-indicator studies, and the largest drawback of such studies is low accuracy. The evolution of IR as a disease is a continuous process involving multiple systems. Therefore, a joint modeling of multiple indicators can effectively increase the accuracy and stability of prediction models and their predictive effects. Rivera et al. (90) found the combination of lipids, cytokines, adipokines, and chemokines, showed a sensitivity and specificity of 93.2% for the identification of IR, which was higher than the effect of a model constructed with a single index. Our research team also constructed an IR risk prediction model based on lipids for obese children, which can be effectively used to predict the risk of IR in obese children (91) (Table 2).

Besides conventional indicators, with the development of technology some new indicators can also be included. Metabolomics can quantify all the metabolic small molecules of organisms and is considered a discipline that can provide the most accurate information based on phenotype (92). Therefore, integrating metabolomics in the IR prediction modeling of obese children, it will most likely be possible to build more stable and accurate models (93, 94). In addition to indicator factors, the method of model construction can also be updated, like machine learning can be used (95, 96).

Although incorporating more indicators or new methods can improve the accuracy of prediction model, the models constructed need to take into account factors such as the simplicity, portability and practicability (97).

## 5 Exercise intervention on insulin resistance

Exercise is key component of energy expenditure and energy balance. It modulates the increase in mitochondrial substrate oxidation in the liver and skeletal muscle, while decoupling this process from pyruvate and acetyl-CoA-driven lipid synthesis (98). According to the recommendations of the WHO, children and adolescents should have at least 60 min of moderate-to-vigorous aerobic exercise per day (99).

**Figure 2:**
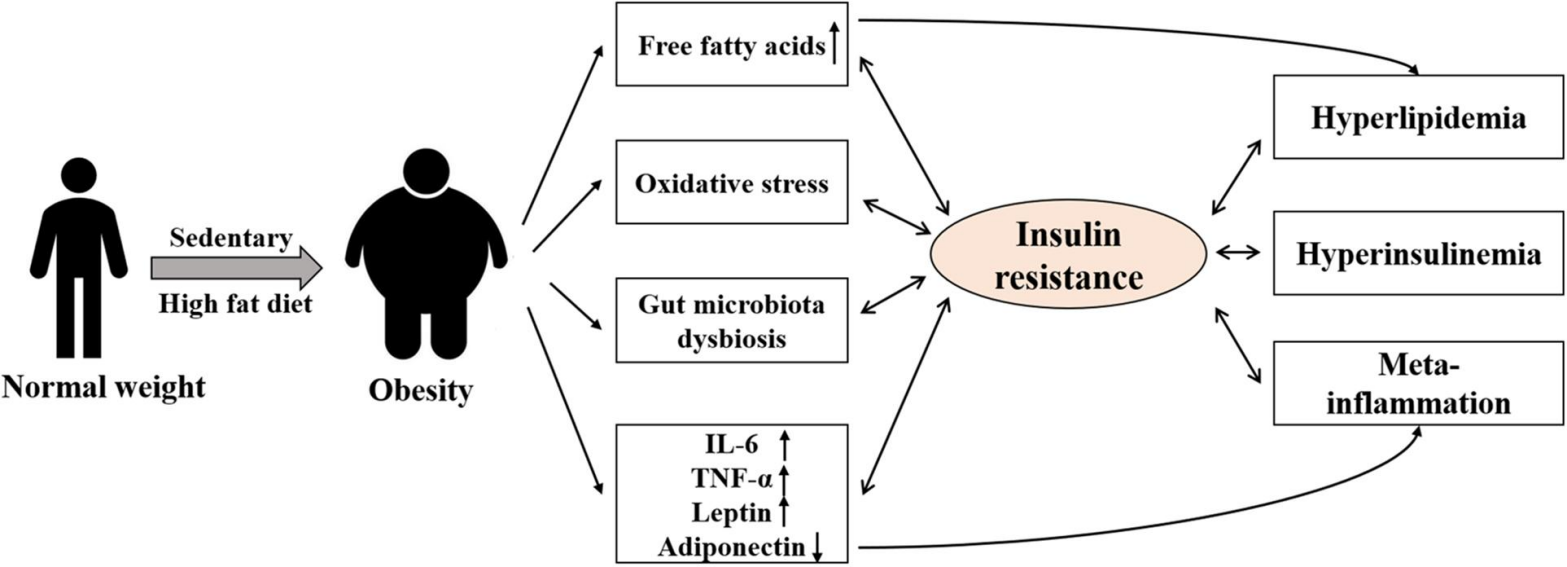
Exercise to improve insulin resistance. Different exercise [aerobic training, high-intensity interval training (HIIT), resistance training, and mixed training] can effective for fat loss, decreased oxidative stress, IL-6, TNF-α and Leptin, ultimately increases insulin sensitivity, which improves IR.

Exercise, an important tool for the prevention and adjunctive treatment of obesity and chronic diseases (100), has a significant effect alleviating lipid metabolism disorders, IR, and insulin sensitivity in obese children and adolescents (101–103). Exercise mainly improves IR by correcting abnormal lipid metabolism (104), decreasing inflammatory response (105–107), increasing glucose transporter protein expression (108), stimulating glycogen uptake (109), and improving the intestinal flora (110). Although exercise can combat IR, no conclusive evidence of the effects of exercise form and intensity or the dose effect of exercise on IR alleviation has been obtained (Figure 2).

### 5.1 Different exercise type

Exercise is usually classified as aerobic training, high-intensity interval training (HIIT), resistance training, and mixed training. It is worth noting that exercise intervention should be tailored to the child's age, pubertal stage, developmental level, comorbidities, and preferences, with gradual progression and appropriate supervision.

Aerobic exercise improves insulin sensitivity mainly by reducing visceral adiposity, decreasing plasma inflammatory factor concentrations (IL-6 and TNF-α) (111), increasing lipocalin levels (112), and increasing GLUT4 expression (113). A meta-analysis of obese children and adolescents found that aerobic exercise significantly improves insulin sensitivity in obese children and adolescents (103). A single session of aerobic exercise is effective in improving insulin sensitivity after exercise but for a short duration (114). By contrast, Lee et al. (101) significantly improved insulin sensitivity in 38 obese or overweight insulin-resistant children and adolescents by subjecting them to long-term aerobic exercise intervention (60 min) three times a week at an intensity of 50%–65% peak oxygen uptake (VO2peak) for 6 months. Abdominal visceral adipose tissue (VAT) is an important determinant of IR across all age groups (115). Increased VAT can cause elevated free fatty acid flux to the liver resulting in IR, aerobic training [treadmill/cycle ergometry at 85% of heart rate maximum (HRmax)] can effectively improved IR by decreasing VAT (116).

HIIT has received considerable interest as a new form of time-saving and efficient exercise. It is more appealing to children and adolescents than prolonged endurance exercise and is more likely to encourage them to participate in exercise (117). HIIT is effective in improving insulin sensitivity and IR in obese children and adolescents (118, 119). However, whether HIIT is more effective than moderate intensity continuous training (MICT) in improving insulin sensitivity is unclear. Corte et al. (118) conducted MICT (30 min, 80% HRmax) and HIIT [1 min 100% maximal oxygen uptake (VO2max) + 3 min 50% VO_2_max, 3–6 cycles] exercise interventions and found that both significantly reduced HOMA-IR, but no significant difference was found. Some studies reported a more pronounced effect of HIIT (120), which may be due to the fact that HIIT increases insulin receptor phosphorylation levels to a higher degree than aerobic exercise (121). Therefore, given that HIIT appeals to children and adolescents, investigating the mechanism of the effect of HIIT in alleviating IR in obese children and adolescents is of great theoretical importance.

Resistance training is widely regarded as the gold standard approach for enhancing muscular fitness in youth while also providing health and physical fitness benefits (122), and resistance training can also effective in improving insulin sensitivity. Lee et al. (101) found that resistance training alone is effective in increasing the rate of glycogen processing in obese children and adolescents after a 60-minute intervention conducted three times a week for 6 months. Resistance training can be equally effective as aerobic training, such as in preventing type 2 diabetes (123). Grøntved et al. (123) found that muscle strength is similarly effective as cardiorespiratory fitness in preserving healthy insulin sensitivity and *β*-cell function later in life, after more than 12 years of tracking research. In addition, evidence suggests that resistance training is more enjoyable than aerobic training (e.g., running, cycling) in children and adolescents, particularly those who are overweight or obese (124, 125).

In conclusion, different types exercise can effectively improve insulin resistance, and studies also found combined exercise may be more effective (126). For obese children and adolescents more enjoyable exercise type is important.

### 5.2 Exercise intensity

Exercise intensity determines the energy substrates during exercise, and the different energy substrates produce different metabolic responses to the body (127). Low to moderate intensity is the optimal intensity for fat oxidation, whereas moderate to high intensity is more significant for carbohydrate consumption (128). Metabolomic studies found that moderate intensity is more beneficial for lipid catabolism than high intensity at the same amount of exercise (129). In resting state, the body's energy supply is dominated by fat oxidation, and phosphorylase activity in skeletal muscles increases with exercise intensity. This effect accelerates glycogen breakdown and results in a gradual increase in the proportion of carbohydrate energy supply (130). Obesity is an important causative factor for IR in the body, and fat elimination is theoretically best facilitated when exercise is performed at maximal fat oxidation (Fatmax) (131), which is the most effective in improving IR while other exercise conditions are controlled. Tan et al. (132) found that a Fatmax exercise intervention performed three times per week 12 weeks, significantly improved insulin sensitivity in subjects. Then whether Fatmax is the optimal intervention intensity for IR needs to be further investigated.

Exercise improves IR by increasing lipid oxidation (104). However, in people with chronic metabolic diseases, such as obesity or T2DM, abnormalities in glycogen to lipid oxidation conversion and decreased lipid oxidation capacity occur, that is, impaired metabolic flexibility (133–135). Resting energy consumption was significantly higher in insulin-resistant than in non-insulin-resistant obese children and adolescents, and the proportion of fat energy supply was significantly lower in insulin-resistant children and adolescents, but the proportion of carbohydrate energy supply was significantly higher (136, 137). Studies on insulin-resistant obese adults found that the maximum rate of fat oxidation during exercise was significantly lower than that of the obese non-insulin-resistant group (137, 138). Moreover, studies on diabetic populations found that the rate of fat oxidation during exercise at 20%, 30%, 40%, 50%, and 60% of maximal aerobic capacity were significantly lower than that of the non-diabetic population (139). This finding suggested significant differences in resting and exercise energy consumption between IR obese children and adolescents and between no-IR obese adolescents and normal-weight children and adolescents.

In conclusion, accurate metabolic characteristics is needed to define exercise intensity criteria of IR obese children and adolescents before develop a personalized exercise prescription.

### 5.3 Exercise dose response

A “dose-effect” relationship was found between the amount of exercise and improved insulin sensitivity. Davis et al. (140) found that high-dose exercise (40 min/day) for 5 days per week is more effective than low-dose exercise (20 min/day) in improving insulin sensitivity and body fat levels in a study of obese children and adolescents that performed the same exercise intensity. Months after the intervention, low-volume and moderate-intensity and high-volume and-high intensity interventions were more effective in improving insulin sensitivity than the low-volume and high-intensity intervention, and the length of the intervention was more important. Exercise dose is composed of exercise intensity, exercise frequency, and exercise duration. This finding suggests that increase in exercise duration increases energy consumption and alleviates lipid metabolism disorders and IR.

No reports related to the minimum dose of exercise for alleviating IR in obese children and adolescents has been published. Nevertheless, on the basis of some studies on insulin sensitivity or diabetes incidence, we can infer that the effect for exercise for alleviating IR has a threshold. Studies on adults found that a single exercise with an energy consumption of 900 kcal effectively improves insulin sensitivity (141); As for long-term exercises, a minimum of 400 kcal of exercise per week significantly improves insulin sensitivity (142).

In addition, the alleviation of IR in obese children may not have a linear relationship with exercise dose. Studies on adults about the association of physical activity with the risk of developing T2DM confirmed this conjecture. Kyu et al. (143) found that the risk of developing T2DM was reduced by 2% when physical activity reached 600 MEs minutes per week compared relative to that in a sedentary and less active population and by another 19% when physical activity was increased to 3,600 METs minutes per week. However, when physical activity was increased from 9,000 METs minutes to 12,000 METs minutes, the risk was reduced by only 0.6%, suggesting that the dose-effect relationship between physical activity and T2DM may not be completely linear.

In conclusion, exercise can improve IR in obese children and adolescents with IR, but the specific quantitative effect relationship needs to be further investigated in combination with the optimal exercise intensity.

## 6 Summary and future directions

IR caused by obesity can induce the occurrence of T2DM, cardiovascular diseases, and cancer, but it is often overlooked because of the absence of obvious physiopathological manifestations in the early stage. Early prevention is far more meaningful than post-treatment. Accordingly assess risk of IR in obese adolescents is absolutely essential, predictive modeling can effectively screen out high-risk factors for the development of IR, so that targeted measures can be taken in subsequent prevention.

Exercise intervention can improve IR, and exercise intensity is closely related to the improvement effect, due to obese children and adolescents with IR have abnormal glucose and lipid metabolism such as metabolic flexibility disorders (144, 145), it is necessary to redefine the exercise intensity standard in people with IR in future studies. In addition, there is a nonlinear dose-response relationship between the improvement effect and the exercise dosage (142), and it is important to explore the specific dose-response relationship for the formulation of precise exercise prescription. Besides programmed exercise interventions, levels of daily physical activity are strongly associated with IR. In future studies, attention should be paid to the level of physical activity of obese children and adolescents, so as to achieve the improvement effect of IR by changing life behaviors. However, it is worth noting that to customized an exercise program requires personalization and should be tailored according to the specific circumstances of the children and adolescents like age, pubertal stage, developmental level, comorbidities, and preferences, with gradual progression and appropriate supervision.

For children and adolescents, a daily dose of safe and vigorous physical activity can be effectively integrated into the school day through daily fitness-focused physical education classes, recess periods, and other structured physical activity opportunities. Schools represent a strategic setting for implementing such public health interventions (146). Exercise interventions with fun, simple games that minimize barriers to participation. Using heart rate or rating of perceived exertion (RPE) as a physiological index of exercise intensity and providing contingent rewards for such exertion, encourage even less-fit children to engage in exercise intense enough to improve metabolic health.
